# The *Drosophila* Microtubule-Associated Protein Mars Stabilizes Mitotic Spindles by Crosslinking Microtubules through Its N-Terminal Region

**DOI:** 10.1371/journal.pone.0060596

**Published:** 2013-04-04

**Authors:** Gang Zhang, Hamze Beati, Jakob Nilsson, Andreas Wodarz

**Affiliations:** 1 Stem Cell Biology, Dept. of Anatomy and Cell Biology, University of Goettingen, Goettingen, Germany; 2 The Novo Nordisk Foundation Center for Protein Research, Faculty of Health Sciences, University of Copenhagen, Copenhagen, Denmark; Institut de Génétique et Développement de Rennes, France

## Abstract

Correct segregation of genetic material relies on proper assembly and maintenance of the mitotic spindle. How the highly dynamic microtubules (MTs) are maintained in stable mitotic spindles is a key question to be answered. Motor and non-motor microtubule associated proteins (MAPs) have been reported to stabilize the dynamic spindle through crosslinking adjacent MTs. Mars, a novel MAP, is essential for the early development of *Drosophila* embryos. Previous studies showed that Mars is required for maintaining an intact mitotic spindle but did not provide a molecular mechanism for this function. Here we show that Mars is able to stabilize the mitotic spindle *in vivo*. Both *in vivo* and *in vitro* data reveal that the N-terminal region of Mars functions in the stabilization of the mitotic spindle by crosslinking adjacent MTs.

## Introduction

Accurate segregation of replicated genomic material relies on the mitotic spindle to coordinate chromosome movements. MTs are assembled from tubulin heterodimers and undergo periods of polymerization and depolymerization, which renders MTs highly dynamic [1]. To keep the stability of such a dynamic MT structure, motor proteins and non-motor MAPs are required to crosslink and stabilize adjacent MTs [2]. Motor proteins like kinesin-5 and kinesin-14 crosslink and slide antiparallel microtubules [3], [4], [5], [6] while the dynein-dynactin complex transports NuMA to the spindle poles to crosslink and focus MT minus ends [7]. PRC1 is a non-motor MAP which bundles interpolar MTs on the central spindle. Depletion of PRC1 affects spindle midzone formation during anaphase and results in failure of cytokinesis [8], [9].

Mars, a novel MAP, is essential for the early development of *Drosophila* embryos [10], [11]. In *mars^91^* mutant embryos, the primary defect is the dissociation of centrosomes from the mitotic spindle and nuclear envelope. Since Mars is not localized on the centrosomes, we reasoned that Mars may be involved in the stabilization of the mitotic spindle. Tan and colleagues discovered that Mars is able to recruit protein phosphatase 1 (PP1) onto the spindle, promoting the dephosphorylation of the *Drosophila* transforming acidic coiled-coil (dTACC) protein [10]. However, TACC has not been reported to possess MT stabilizing activity on its own [12]. How Mars contributes to the stabilization of the mitotic spindle is still unclear.

Here we show that Mars is able to stabilize mitotic spindles *in vivo*. This function is mainly mediated by its N-terminal region (aa 1–430). *In vitro* studies show that an MBP fusion protein with the N-terminal half of Mars (MBP-N-Mars) is able to bind to MTs and to stimulate the crosslinking and bundling of MTs. Overexpression of the N-terminal half of Mars (GFP-N-Mars) in *Drosophila* embryos caused narrowing of spindle poles and fusion of mitotic spindles, while overexpression of the C-terminal half of Mars (GFP-C-Mars) impaired proper assembly of the mitotic spindle and caused nuclear fusion. Rescue assays show that neither GFP-N-Mars nor GFP-C-Mars can rescue the lethality of *mars^91^* mutant embryos. However, GFP-N-Mars is able to largely maintain the mitotic spindle morphology in the absence of endogenous Mars. The failure of GFP-N-Mars to rescue the embryonic lethality of the *mars^91^* mutation is probably due to its excess of MT bundling activity, which may cause spindle fusion or narrowing of spindle poles, leading to detachment of centrosomes from the main spindle.

## Materials and Methods

### Generation of transgenic fly lines

Transgenic flies were generated using the phiC31 integrase system [13]. Briefly, plasmids of pUASP-GFP-Mars, pUASP-GFP-N-Mars (aa 1-430) and pUASP-GFP-C Mars (aa 431–921), all carrying an attB site, were injected into the posterior ends of embryos carrying an attP landing site at position 99F8 on the third chromosome (Bloomington stock #9738) with a micromanipulator (InjectMan NI2, Eppendorf). After injection, embryos were kept in 10 S Voltalef oil at 18°C for 48 hr before the hatched larvae were collected.

### *in vivo* mitotic spindle stabilization assay

0–4 hr old embryos were dechorionated in 50% bleach and rinsed with embryo washing buffer (0.7% NaCl, 0.03% Triton X-100). The embryos were resuspended in Schneider's medium without antibiotics while containing 0.15 µM of demecolcine (D7385, Sigma). 3 ml of n-heptane was added and the suspension was vigorously vortexed for 30 sec before shaking at 50 rpm for 20 min on the shaking plate. The medium was removed after the treatment and 3 ml of strong fixation solution was added to fix the embryos. The fixation was done for 5 min by shaking on the plate. Fixation solution was removed and 3 ml of methanol was added. The mixture was vortexed strongly for 30 sec. All the embryos that sank to the bottom of the vial were collected and washed another three times with methanol. The fixed embryos were kept at −20°C till use.

### Immunofluorescence

Strong fixation was used in this study as described before [11]. Immunofluorescence was done according to standard procedures [14]. The antibodies used for immunofluorescence were rabbit anti Mars (1:200), mouse anti tubulin E7 (1:50; Developmental Studies Hybridoma Bank, DSHB) and mouse anti tubulin-FITC (Sigma). DNA was stained with DAPI. Images were taken on a Zeiss LSM510 confocal microscope (Carl Zeiss, Germany) or on a DeltaVision fluorescence microscope (Applied Precision, US). Spindle width and length measurements were performed with LSM510 Meta software (Carl Zeiss).

### Recombinant protein purification

The overnight bacterial culture was diluted 1:100 in 2 liter of pre-warmed LB medium. Induction was performed when the OD_600_ reached 1.0 by 0.2 mM IPTG at 20°C. The induction was continued overnight. Bacteria were harvested by centrifugation at 7,700 × g for 10 min and resuspended in 7.5 ml of lysis buffer containing 80 mM HEPES, pH 7.0, 100 mM NaCl, 2 mM MgCl_2_ and 0.5 mM EGTA. The bacteria were lysed with a high-pressure homogenizer (Avestin). The lysate was spun down at 12,000 × g for 15 min before incubating with 2 ml of amylase beads at room temperature for 30 min. The fusion protein was eluted from the beads with 10 mM maltose in lysis buffer. 1 ml of eluted protein was diluted with 4 ml of 20 mM HEPES buffer, pH 7.8, containing 50 mM NaCl, 0.5 mM DTT and loaded onto a HiTrap SP FF column (GE Healthcare). The protein was further purified with an AKTA chromatography system (GE Healthcare). Purified protein was desalted and concentrated with PEM buffer (80 mM PIPES, pH 6.9, 1 mM MgCl_2_, 1 mM EGTA) with 10 mM NaCl by centrifugal filter (Amicon Ultra 0.5, Millipore).

### MT binding assay

20 mg/ml tubulin solution was prepared in PEM with 13.3% glycerol and 1 mM GDP-NP. The solution was incubated at 35°C for 20 min. MTs were stabilized by addition of 5 mM taxol with another 10 min incubation at 35°C. A final concentration of 7.5 µM of MT solution was prepared in the same buffer supplemented with 150 mM NaCl, 1 mM BSA and 100 µM taxol. 100 µl of cushion buffer (PEM with 50% glycerol) supplemented with 100 µM of taxol and 1 mM of GDP-NP was transferred into labeled thick wall tubes. The binding reaction was set up by mixing the MT solution with MBP-N-Mars protein. The reaction was kept at room temperature for 5 min before being loaded onto the cushion buffer. Ultracentrifugation at 100,000 x g was performed for 20 min at 25°C. 20 µl of supernatant from the top was taken and mixed with 6.6 µl 4 × SDS loading buffer. The pellet was resuspended in 25 µl of 2 × SDS loading buffer. Both supernatant and pellet samples were analyzed by SDS-PAGE and Coomassie Brilliant Blue staining.

### MT polymerization assay

The assay was modified as described elsewhere [15]. Briefly, 20 µM tubulin stock solution in PEM buffer containing 1 mM GTP and 10% glycerol was prepared and put on ice for 30 mins. 5 µl of taxol (30 µM) and protein samples (2.5 µM) were transferred into wells of a 37°C pre-warmed 96-well plate. 45 µl of 20 µM tubulin stock was added into each well quickly. The OD at 340 nm was read immediately for 30 cycles of 1 reading/min.

### Microtubule stabilization assay

The microtubule stabilization assay was performed according to a published method [16]. Briefly, preassembled microtubule (50 µM) was incubated with or without recombinant MBP-N-Mars (5 µM) at 35°C for 30 min. The mixture was diluted to different times and further incubated at 35°C for another 30 min before being centrifuged at 100,000 × g 30 min. The pellet was resuspended in 50 µl 1 × sample loading dye and analyzed by SDS-PAGE. The final quanitification was done with ODYSSEY Sa system (LI-COR).

### *in vitro* MT bundling assay

The assay was conducted as described elsewhere [15]. Briefly, 20 µl of tubulin polymerization reaction was set up in PEM buffer containing 10% glycerol, 100 µM of tubulin and 1 mM GTP. The mixture was incubated at 37°C for 30 min. 180 µl of pre-warmed BRB80 10% buffer containing 20 µM taxol was added into the mixture and incubated at 37°C for 10 min. 10 µl of taxol-stabilized MTs was mixed with 10 µl of MBP (2.5 µM) or MBP-N-Mars (2.5 µM). The mixture was incubated at 37°C for 15 min before being dropped onto a poly-L-lysine-treated glass slide (Sigma) for 5 min. 4% paraformaldehyde in PBS was used for fixation for 30 min at room temperature. The slide was pre-blocked with 8% BSA in PBS for 30 min, followed by incubation with anti alpha-tubulin-FITC antibody overnight at 4°C. The slide was washed three times with PBS and analyzed by fluorescence microscopy (Deltavision, Applied Precision).

### *in vitro* α/β-tubulin heterodimer binding assay

The assay was performed according to the protocol described elsewhere [17]. 0.625 µg of MBP or MBP-fusion protein was mixed with 30 µl 0.5 mg/ml tubulin solution in PEM buffer. The mixture was incubated on ice for 30 min. 50 µl of amylase beads were added and incubated by shaking at 4°C for 30 min. Beads were washed four times with 1 ml PEM buffer. Bound protein was eluted by 20 mM maltose in PEM and analyzed by Western blot.

## Results

### Mars is able to stabilize mitotic spindles at the syncytial blastoderm stage of *Drosophila* embryos

**Figure 1:**
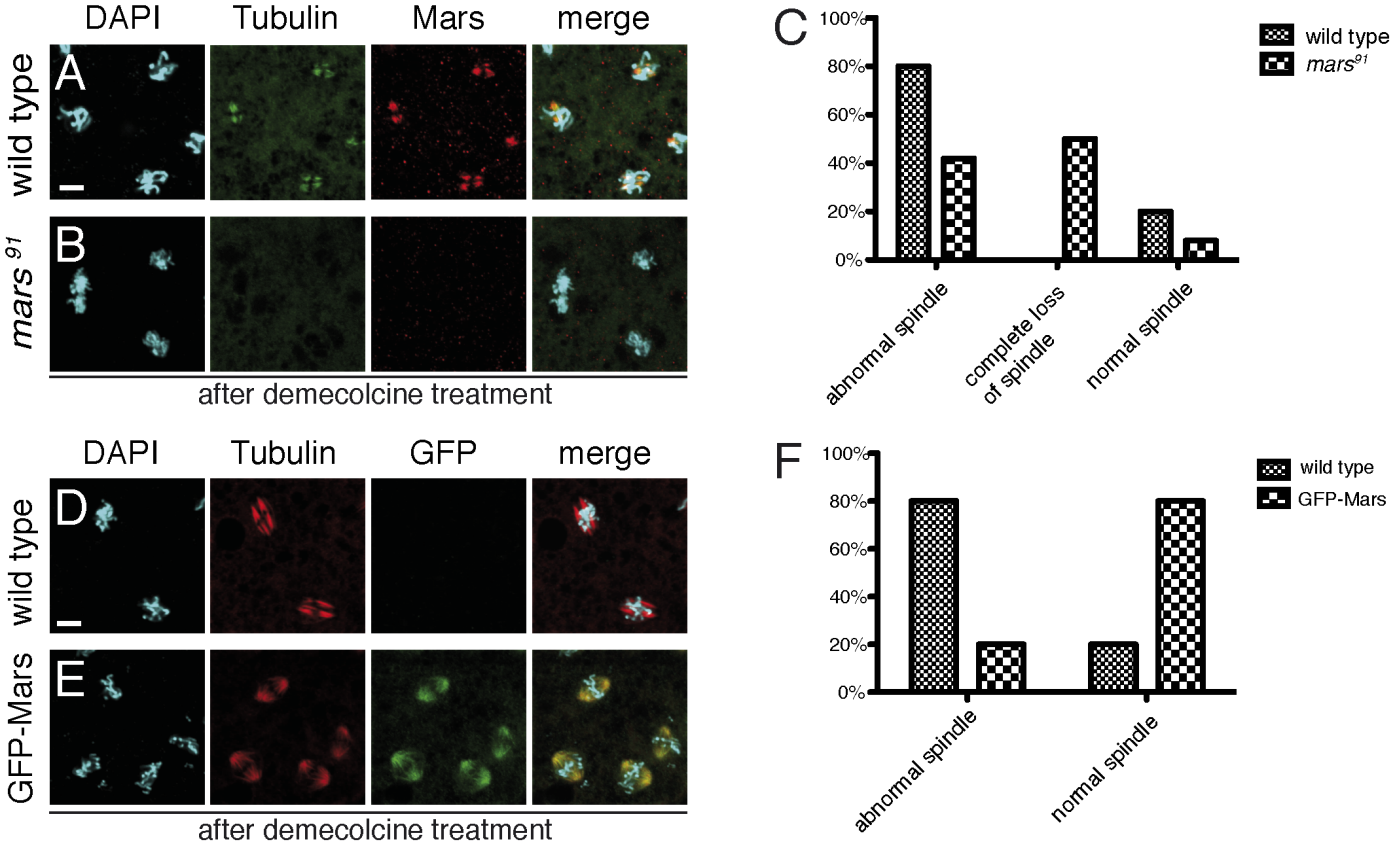
Mars stabilizes the mitotic spindle *in vivo*. (A, B) Wild type (A) and *mars^91^* mutant embryos (B) were mixed and treated with 0.15 µM demecolcine to destabilize mitotic spindles. Embryos were fixed and stained by tubulin antibody (green), Mars antibody (red) and DAPI (turquoise). (C) Quantification of spindle phenotypes for the genotypes shown in (A, B). (D, E) Wild type embryos (D) and embryos overexpressing GFP-Mars driven by daughterless>Gal4 (E) were mixed and treated like in (A, B). Embryos were fixed and stained by tubulin antibody (red), GFP antibody (green) and DAPI (turquoise). (F) Quantification of spindle phenotypes for the genotypes shown in (D, E). For the quantifications in (C) and (F), 200 spindles from 10 embryos were scored for each genotype. Scale bar is 5 µm.

In our previous study we showed that Mars is essential for the early development of *Drosophila* [11]. *Drosophila* embryos lacking maternal and zygotic Mars showed a spectrum of defects including aberrant mitotic spindle morphology, centrosome detachment, increased formation of MT asters and failed chromosome segregation. We hypothesized that Mars may be involved in the structural stabilization of the mitotic spindle. To test this hypothesis, we performed *in vivo* mitotic spindle stabilization assays. Wild type embryos and *mars^91^* mutant embryos were mixed and treated with a low concentration of demecolcine, which partially depolymerized MTs from the mitotic spindles. In wild type embryos, around 80% of mitotic spindles were of abnormal morphology with split spindle poles and MT loss in between kinetochore fibers (Fig 1A, C). In *mars^91^* mutant embryos, the defects were significantly enhanced. Around 50% of *mars^91^* embryos completely lost MTs around the chromosomes (Fig 1B, C) while the remaining embryos showed much weaker staining of MTs (data not shown). Obviously, *mars* mutant embryos were much less resistant to the MT depolymerizing drug. We next asked if overexpression of Mars could enhance the resistance of the mitotic spindles to depolymerization. Embryos with a mild overexpression of GFP-Mars driven by GAL4 under control of the ubiquitous *daughterless* promoter were mixed with wild type embryos and treated with demecolcine as described above. Around 80% of the embryos with overexpressed GFP-Mars showed normal morphology of the mitotic spindles with focused spindle poles and even distribution of MTs (Fig 1E, F), whereas only around 20% of wild type embryos had normal spindles (Fig 1F). The remainder of the wild type embryos showed similar defects as described above (Fig 1D, F). These data confirm our hypothesis that Mars is involved in the stabilization of MTs within mitotic spindles.

### The N-terminal region of Mars binds MTs directly and stimulates MT assembly through crosslinking of adjacent MTs

**Figure 2:**
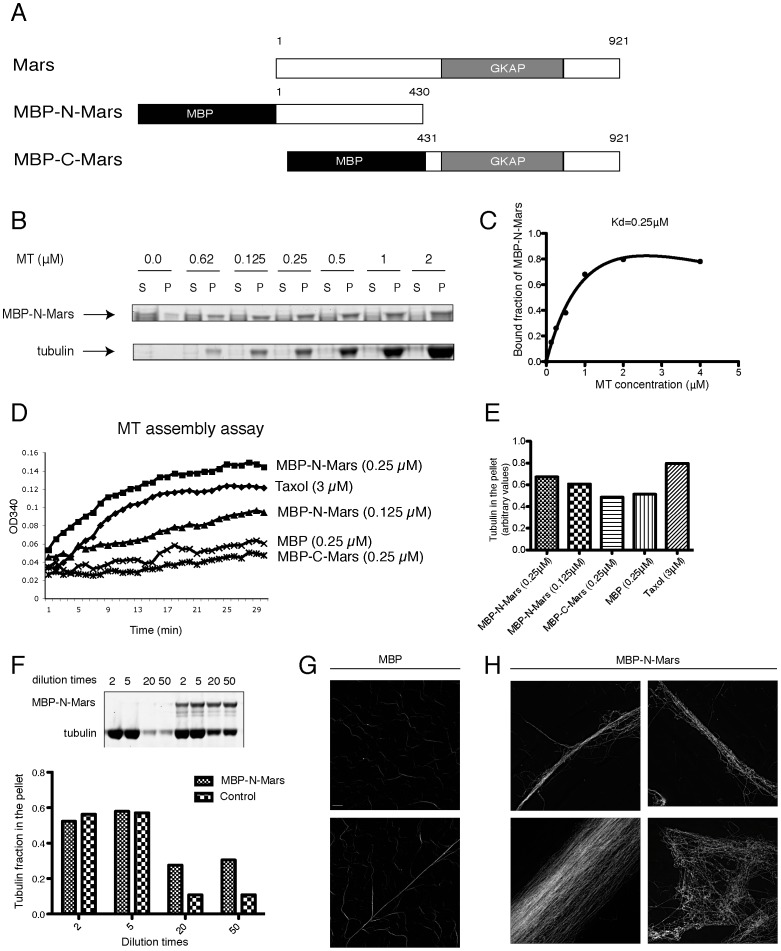
MBP-N Mars binds and stabilizes microtubules in vitro. (A) Scheme of recombinant proteins used in this study. (B) MBP-N-Mars binds to MTs *in vitro*. 1 µg of purified MBP-N-Mars was incubated with taxol-stabilized MTs at different concentrations before ultracentrifugation through a glycerol cushion. (C) Plot of bound fraction of MBP-N-Mars against MT concentration. The calculated Kd value is given above the curve. (D) MBP-N-Mars stimulates the assembly of MTs. The indicated amounts of MBP protein, MBP-N-Mars and MBP-C-Mars were incubated with tubulin solution. Taxol was used as a positive control. (E) Quantification of the tubulin in the pellet after sedimentation of the samples according to the same procedure as in (D). (F) MT dilution assay in the absence or presence of MBP-N-Mars. Top: Coomassie Brilliant Blue staining of the pellet samples separated by SDS-PAGE. Bottom: Quantification of the Coomassie staining results by LI-COR ODYSSEY SA system. (G, H) Microtubule bundling assay. The same amount of MBP (G) and MBP-N-Mars (H) was incubated with tubulin solution in the presence of a low concentration of taxol. Tubulin structures were fixed by formaldehyde, stained with tubulin-FITC antibody and imaged by fluorescence microscopy. Scale bar is 5 µm.

In our previous study, we found that the N-terminal half of Mars (N-Mars) is required and sufficient for the localization of Mars on mitotic spindles *in vivo* [11]. Is the binding of N-Mars to the mitotic spindle involved in the stabilization of the spindle? To answer this question, we generated constructs encoding MBP tagged truncated versions of Mars (Fig 2A) and purified recombinant proteins by affinity chromatography on amylose beads and subsequent cation exchange chromatography. We then performed *in vitro* MT binding and stabilization assays with MBP-N-Mars. From the MT binding assay, MBP-N-Mars was found to bind taxol-stabilized MTs (Fig 2B). The bound protein fraction was plotted against the MT concentration, which gave a hyperbolic curve similar to other MT binding proteins (Fig 2C) [15]. From this curve we calculated a Kd value of 0.25 µM for binding of MBP-N-Mars to MTs. MBP-C Mars did not show a specific binding to MTs (data not shown).

To test whether the binding has any effect on the stability of MTs, we performed a MT assembly assay. We found that MBP-N-Mars stimulated the assembly of MTs in a dose dependent manner (Fig 2D). Compared with the known MT stabilizer, taxol, the stimulation was much faster and more efficient even at a much lower concentration (0.25 µM of MBP-N-Mars vs 3 µM of taxol). MBP-C-Mars was also tested in this assay and showed an OD_340_ profile comparable to that of MBP protein, indicating that MBP-C-Mars cannot stimulate the assembly of MTs. To test whether the increase in the turbidity at 340 nm may eventually be caused by MT bundling, we performed a sedimentation assay after the assembly of MTs. In this assay, MBP-N-Mars (0.25 µM) was less efficient in sedimentation of tubulin than taxol. This finding points to a contribution of MT bundling to the high turbidity reading caused by incubation of MTs with MBP-N-Mars. However, 0.25 µM of MBP-N-Mars sedimented more MTs than 0.125 µM of N-Mars and 0.25 µM of C-Mars and MBP.

To further investigate whether the binding of MBP-N-Mars is able to stabilize MTs, we performed a MT dilution assay. Pre-assembled MTs were diluted in the absence or presence of MBP-N-Mars followed by a sedimentation assay. The tubulin in the pellet was analyzed by SDS-PAGE (Fig 2F). At low dilution factors (2x and 5x) the addition of MBP-N-Mars had no effect on the stability of MTs (Fig 2F). At higher dilution factors (20x and 50x) however, addition of MBP-N-Mars increased the amount of tubulin in the pellet by a factor of three (Fig 2F). This result provides additional evidence for MBP-N-Mars being able to stabilize MTs *in vitro*.

To further investigate the details of MT stabilization by MBP-N-Mars, we did a MT bundling assay and observed the MTs by indirect immunofluorescence. MBP or MBP-N Mars was mixed with tubulin at 1:4 molar ratio. The mixture was incubated at 37°C for 15 min, followed by formaldehyde fixation and tubulin antibody staining. By fluorescence microscopy, numerous short thin MT fibers were found in the sample with MBP protein (Fig 2G, upper panel). Occasionally, a few long thin fibers were found (Fig 2G, lower panel). By contrast, in the sample incubated with MBP-N-Mars, very few short and thin fibers were detected. Instead, long thick MT fibers with different widths were frequently observed (Fig 2H, left and top right panels). Besides these thick fibers, another type of MT structure resembling a network was also found (Fig 2H, bottom right panel). These data strongly indicate that N-Mars stabilizes MTs by crosslinking them.

### Overexpression of GFP-N Mars leads to pointed spindle poles *in vivo*

**Figure 3:**
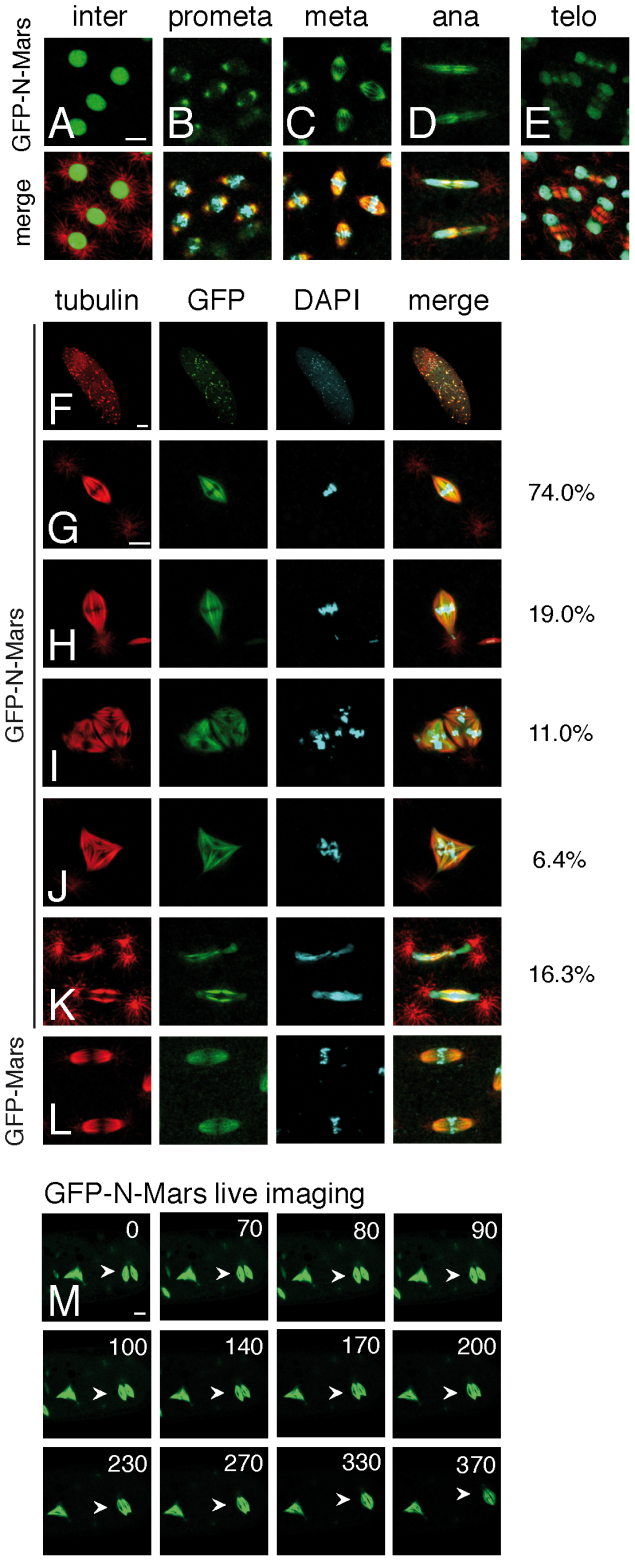
Overexpression of GFP-N Mars causes embryonic lethality and mitotic spindle defects. (A–E) Subcellular localization of GFP-N-Mars in transgenic fly embryos. Embryos were fixed and stained by tubulin antibody (red), GFP antibody (green) and DAPI (turquoise). Scale bar is 5 µm. (F–K) Overexpression of GFP-N-Mars causes embryonic lethality and defects in mitotic spindle morphology. (F) Overview of GFP-N-Mars overexpressing embryo showing abnormal pattern of early mitoses. (G) Acentrosomal spindle with pointed spindle poles. (H) Chromosome lagging at spindle pole. (I) Attached multipolar spindles. (J) Tripolar spindle. (K) Chromosome segregation failure with chromosomal bridges. Numbers to the right of panels (G–K) indicate the frequency of the respective phenotypes. Numbers add up to more than 100% since some spindles show a combination of two or more abnormalities. 300 mitotic spindles from 15 embryos were scored. (L) Spindles from control embryo overexpressing GFP-Mars show normal morphology. Embryos were fixed and stained by antibodies described in (A–E). Scale bars are 40 µm for panel (F) and 5 µm for panels (G–L). (M) Still images from live imaging of GFP-N-Mars showing fusion of two mitotic spindles (cf. Movie S2). Scale bar is 10 µm.

To confirm *in vivo* what we found *in vitro*, a GFP-N-Mars (aa 1–431) transgenic fly line was generated. The subcellular localization of GFP-N-Mars was recorded from fixed or live embryos by indirect immunofluorescence or live imaging (Fig 3; Movie S1). Very similar to the localization of full length Mars (GFP tagged or endogenous Mars) [11], GFP-N Mars was in the nucleus at interphase (Fig 3A) and on the mitotic spindle at metaphase (Fig 3C). Similar to full length Mars, GFP-N-Mars was not detected at centrosomes or centrosomal microtubules (Fig 3 and data not shown). However, different to full length Mars, GFP-N-Mars showed prominent localization on the central spindle at anaphase instead of being restricted to spindle poles (Fig 3D) [11]. This finding indicates that the C-terminal region may be responsible for the exclusion of Mars from the central spindle during anaphase.

In contrast to overexpression of full length GFP-Mars (Fig 3L), overexpression of GFP-N-Mars by maternally provided GAL4 caused lethality to embryos. Only 2.8% of embryos hatched as larvae while the remaining 97.2% died during embryogenesis. In individual mitotic spindles, three highly correlated major defects were observed. First, centrosomes often dissociated from mitotic spindles, which is similar to the defect found in *mars^91^* mutant embryos (Fig 3G) [11]. Surprisingly, the spindle poles were always sharply focused, even in cases where the centrosomes detached from the spindle. Second, the mitotic spindles without centrosomes tended to stack together instead of collapsing into monopolar spindles as in *mars^91^* mutant embryos (Fig 3I, M) [11]. Very few monopolar mitotic figures were found in embryos overexpressing GFP-N-Mars, whereas in *mars^91^* mutant embryos 30% of mitotic figures were monopolar. Figures of three mitotic spindles were often observed to stack together and sometimes fused into a tripolar mitotic structure (Fig 3I, J). By live imaging, we recorded the fusion of two single mitotic spindles into one bipolar spindle (Fig 3M; Movie S2). The two spindles were arranged in parallel to each other at the beginning. Then the middle part of both spindles started to touch and becoming connected. After around 5 min, a wider bipolar spindle was formed (Fig 3M; Movie S2). Third, single chromosomes were also found at the spindle poles while the remaining chromosomes were aligned at the metaphase plate (Fig 3H). Chromosome segregation was also impaired in some cases where chromosome bridges formed in between the spindle poles at anaphase (Fig 3K).

Can overexpression of GFP-N-Mars enhance the stability of the mitotic spindle? We performed an *in vivo* MT stabilization assay using the MT depolymerizing drug demecolcine as described above. As expected, in the presence of overexpressed GFP-N-Mars, most of the mitotic spindles kept the poles focused and showed a relatively even distribution of MTs within the spindles (Fig S1).

### Overexpression of GFP-C-Mars impairs the assembly of the mitotic spindle *in vivo*

**Figure 4:**
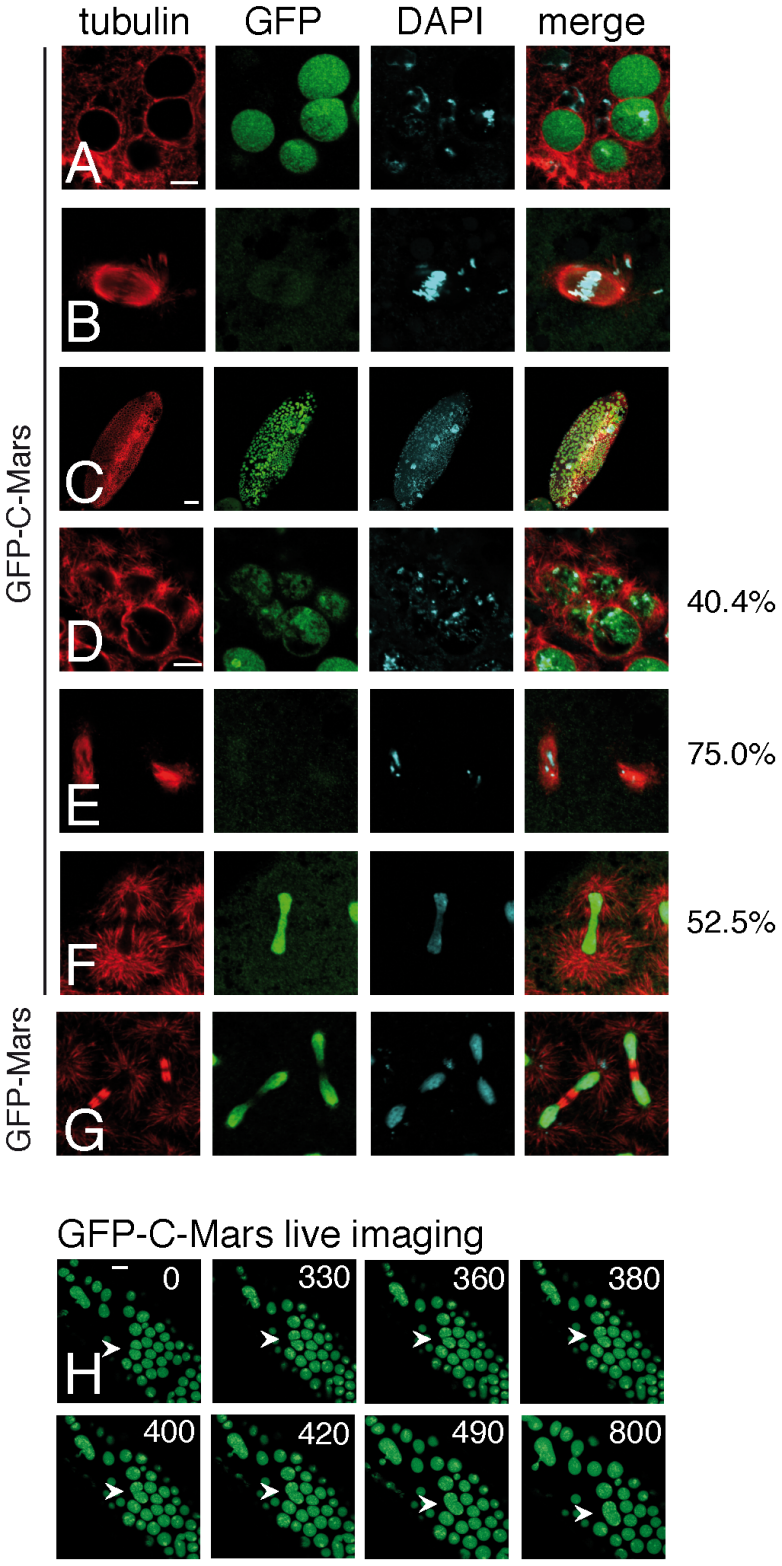
Overexpression of GFP-C Mars causes embryonic lethality and impairs the assembly of the mitotic spindle. (A, B) Subcellular localization of GFP-C-Mars at interphase (A) and metaphase (B). (C–F) Defects caused by overexpression of GFP-C-Mars in embryos. (C) Overview of GFP-C-Mars overexpressing embryo showing abnormal pattern of early mitoses. (D) Giant nuclei. (E) Poorly organized mitotic spindles. (F) Excessive formation of astral microtubules. Numbers to the right of panels (D–F) indicate the frequency of the respective phenotypes. Numbers add up to more than 100% since some spindles show a combination of two or more abnormalities. 200 mitotic spindles from 10 embryos were scored. (G) Spindles from control embryo overexpressing GFP-Mars show normal morphology. Embryos were fixed and stained with tubulin antibody (red), GFP antibody (green) and DAPI (turquoise). Scale bars are 40 µm in panel (C) and 5 µm in panels (A, B, D–G). (H) Still images from live imaging of GFP-C-Mars showing fusion of two nuclei (cf. Movie S4). Scale bar is 20 µm.

Our data point to a function of the C-terminal region of Mars in the proper localization and functioning of the full-length protein. The *in vitro* data furthermore did not reveal an effect of the C-terminal region of Mars on MT assembly (Fig 2D, E). To investigate the *in vivo* roles of the C-terminal region, we generated a GFP-C-Mars (aa 431–921) transgenic fly line. GFP-C-Mars localized to the nucleus at interphase as reported previously [11] (Fig 4A, Movie S3). At metaphase, it was dispersed in the whole cytoplasm (Fig 4B, Movie S3). Overexpression of GFP-C-Mars caused severe lethality to embryos with a hatching rate of only 8.5%. Different to the defects caused by overexpression of GFP-N-Mars, giant nuclei and poorly organized mitotic spindles were the most common defects observed (Fig 4D, E). The giant nuclei could be the result of failed segregation of duplicated chromosomes as shown in Fig 4F. However, by live imaging we observed another way for the generation of this type of nuclei. We observed two nuclei touching each other and then fusing into one big nucleus (Fig 4H, Movie S4). When GFP-C-Mars was recruited back into the nucleus at telophase, astral MTs formed in excess around the centrosomes (Fig 4F), in contrast to telophase spindles of embryos overexpressing full length GFP-Mars (Fig 4G).

### GFP-N Mars competes with endogenous Mars for localization in nuclei and on the mitotic spindle

**Figure 5:**
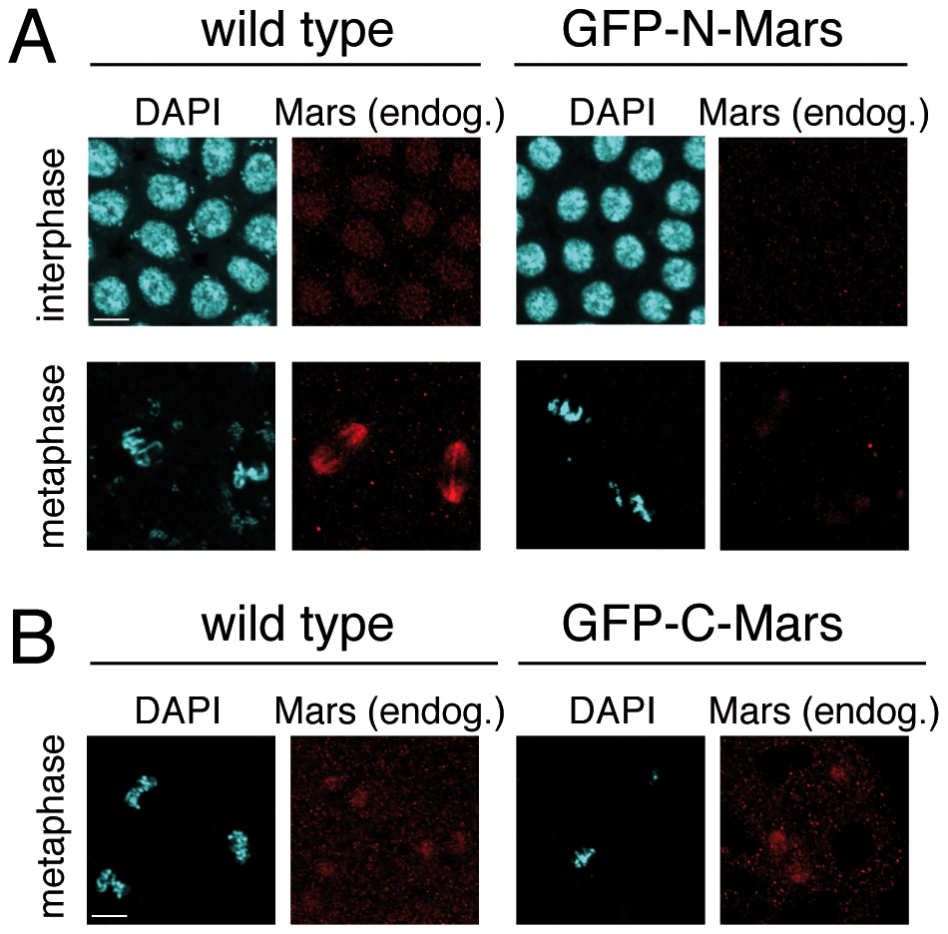
Overexpression of GFP-N-Mars causes reduced localization of endogenous Mars in the nucleus and on the mitotic spindle. (A) Wild type embryos and embryos overexpressing GFP-N-Mars were fixed and stained by GFP antibody, Mars antibody raised against the C-terminus (red) and DAPI (turquoise). (B) Wild type embryos and embryos overexpressing GFP-C-Mars were fixed and stained by GFP antibody, Mars antibody raised against the N-terminus (red) and DAPI (turquoise). GFP channels not shown. Scale bar is 5 µm.

To investigate potential effects on endogenous Mars when the truncated Mars proteins were overexpressed in embryos, we used antibodies raised against either the N-terminus or the C-terminus to distinguish endogenous Mars from overexpressed GFP-N-Mars or GFP-C-Mars. In stainings with the antibody specific to the C-terminus, the signals from endogenous Mars were much fainter in the nuclei and on the mitotic spindle in embryos overexpressing GFP-N-Mars compared with the signals from wild type embryos under the same imaging conditions (Fig 5A). These data indicate that GFP-N-Mars may compete with endogenous Mars for binding to importins and to mitotic spindle MTs. A similar experiment was performed with antibodies specific to the N-terminus. However, this antibody did not recognize Mars in nuclei. On mitotic spindles, only faint signals were detected with rather high background (Fig 5B). We could not detect a significant difference with respect to staining intensity and subcellular localization of endogenous Mars between wild type embryos and embryos overexpressing GFP-C-Mars.

### GFP-N-Mars partially rescues *mars^91^* spindle defects but fails to rescue embryo lethality

**Figure 6:**
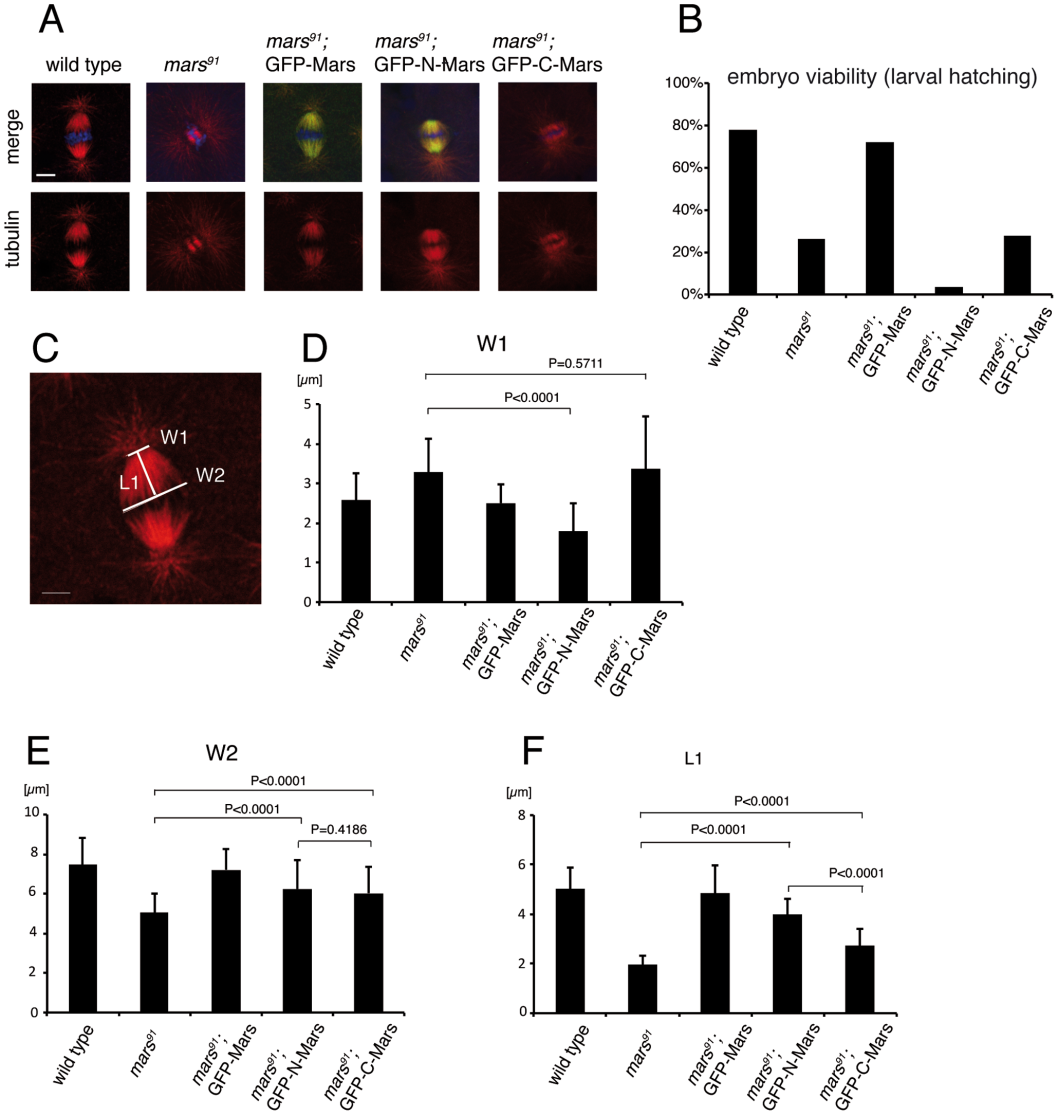
Rescue of *mars^91^* mutant phenotypes by GFP-N-Mars and GFP-C-Mars. (A) Typical mitotic spindles in wild type and *mars^91^* mutant embryos upon expression of GFP-Mars, GFP-N-Mars or GFP-C-Mars. Embryos were fixed and stained with tubulin antibody (red), GFP antibody (green) and DAPI (turquoise). Scale bar is 5 µm. (B) Quantification of larval hatching of wild type embryos, *mars^91^* mutant embryos and *mars^91^* mutant embryos rescued by the respective transgenes. (C) Illustration of spindle parameters quantified in (D–F). (D–F) Quantification of mitotic spindle parameters from wild type embryos, *mars^91^* mutant embryos and *mars^91^* mutant embryos rescued by GFP-Mars, GFP-N-Mars or GFP-C-Mars. Embryos analyzed in (C–F) were fixed and stained by tubulin antibody (red). Scale bar in (C) is 5 µm. t-test was performed by Prism 5 (GraphPad Software).

To further investigate the functional properties of GFP-N-Mars and GFP-C-Mars, we performed rescue assays. In these experiments, wild type full-length Mars and both truncated forms of Mars were expressed in the *mars^91^* mutant background (Fig 6A). *mars^91^* carries a deletion of 531 bp including the ATG start codon and represents a null allele of *mars* [11]. Full-length Mars largely rescued the lethality of *mars^91^* mutant embryos (80% hatching rate of embryos; Fig 6B). Expression of GFP-C-Mars in the *mars^91^* mutant background resulted in hatching rates close to the *mars^91^* mutant without transgene (Fig 6B), demonstrating that GFP-C-Mars did not possess any rescuing activity. In contrast to our expectation, GFP-N-Mars expression lowered the survival rate of *mars^91^* mutant embryos (3.4% vs 26.4%, Fig 6B), which points to a dominant-negative effect of GFP-N-Mars expression. This effect may be due to the fact that GFP-N-Mars expression causes excess bundling of MTs at spindle poles, which may affect the interaction between spindle poles and centrosomes. However, close examination of individual mitotic spindles showed a certain extent of rescue with respect to spindle morphology by GFP-N-Mars. We analyzed the mitotic spindle morphology by measuring the width of the spindle close to the chromosomes (W2) and close to the centrosomes (W1) and also the length of the half spindle (L1) (Fig 6C). t-test showed a significant difference of W1 between the *mars^91^* mutant and the *mars^91^* mutant rescued by GFP-N-Mars expression (Fig 6D). By contrast, no significant difference of W1 was observed between the *mars^91^* mutant and the *mars^91^* mutant rescued by GFP-C-Mars (Fig 6D). For the length of the half spindle, L1, GFP-N-Mars overexpression led to a significant increase compared to the *mars^91^* mutant and GFP-C-Mars overexpression (Fig 6F).

## Discussion

Our previous study showed that *Drosophila* Mars is essential for the integrity of the mitotic spindle during early embryogenesis [11]. Here we performed *in vitro* biochemical assays and *in vivo* genetic overexpression studies to investigate in detail the function of Mars. Our data clearly show that a fusion protein of MBP with the N-terminal region of Mars (MBP-N-Mars) is able to stabilize MTs by crosslinking adjacent MTs *in vitro*. Overexpression of GFP-N-Mars *in vivo* enhanced the resistance of mitotic spindles towards a MT depolymerizing drug, resulted in narrower spindle poles and impaired the dynamics of mitotic spindles, which confirms the MT stabilization function found *in vitro*. Upon overexpressing GFP-N-Mars, dissociation of centrosomes from mitotic spindles was often observed. Although we also observed a significant loss of endogenous Mars from mitotic spindles upon overexpression of GFP-N-Mars, we do not think that this is the reason for centrosome dissociation. Our data indicate that in *mars* mutant embryos, the mitotic spindles are not as stable as in wild type embryos. Once tension is generated, the centrosomes begin to dissociate from the mitotic spindle. However, in embryos overexpressing GFP-N-Mars, the mitotic spindles are very stable as shown by the in *vivo* mitotic spindle stabilization assay. This view is supported by the rare occurrence of monopolar spindles upon overexpression of GFP-N Mars, whereas more than 30% of the mitotic spindles from *mars* mutant embryos were monopolar. Because overexpression of GFP-N-Mars narrowed the spindle poles, it is likely that this effect reduced the area of interaction between spindle poles and centrosomes, resulting in the dissociation of centrosomes from the spindle.

By live imaging, we recorded the fusion of a pair of spindles in an embryo overexpressing GFP-N-Mars. Considering the ability of MBP-N-Mars to cross-link MTs *in vitro*, the mitotic spindle fusion may be caused by crosslinking MTs from adjacent spindles. From immunofluorescence studies of fixed embryos, we found that more than 10% of the spindles were tripolar. Obviously not all fusions events result in bipolar spindles, which can be explained by the geometry of the spindles when they first touch each other. A recent study on spindle fusion [18] proposed that dynein-dependent pulling forces on overlapping polar MT arrays could drive spindle fusion in *Xenopus* eggs. Whether dynein is also involved in the spindle fusions observed upon GFP-N-Mars overexpression remains to be investigated.

Overexpression of GFP-C-Mars in *Drosophila* embryos resulted in poorly organized mitotic spindles with lower MT density and unfocussed poles. The mechanism responsible for this phenotype is not clear at the moment. We found that both MBP-N-Mars and MBP-C-Mars can bind alpha/beta tubulin heterodimers *in vitro* (data not shown). Apparently, C-Mars shows a higher binding affinity towards tubulin heterodimers. Since C-Mars does not show MT binding ability *in vivo*, a high amount of GFP-C-Mars in the cytoplasm may sequester the tubulin heterodimers and interfere with the proper assembly of the mitotic spindle.

We noticed that GFP-N-Mars localized to the central spindle during anaphase, which is not the case for full length GFP-Mars, pointing to a function of the C-terminal region of Mars in preventing localization to the central spindle. At least 9 *in vivo* phosphorylation sites of Mars are known in the region spanning aa 431–921 [19], [20]. Mutation of some of these serine/threonine residues to alanine results in enhanced localization of the mutant full-length GFP-Mars protein to the central spindle (GZ, unpublished data). From these findings we conclude that phosphorylation of specific sites in the C-terminal region of Mars regulates the subcellular localization of full length Mars.

Tan and colleagues reported one PP1 binding site within the C-terminal region of Mars. The binding promoted the dephosphorylation of dTACC [10]. How dephosphorylated dTACC could contribute to mitotic spindle stability is not clear yet. This raises the question as to the main function of Mars, MT stabilization through the N-terminal region or the promotion of dephosphorylation of dTACC through the C-terminal region? Our results show that the N-terminal region of Mars is able to stabilize MTs both *in vitro* and *in vivo*. Though expression of GFP-N-Mars alone could not rescue the lethality of the *mars^91^* mutant, the defective mitotic spindle morphology observed in *mars^91^* was largely rescued. We conclude that with respect to the function of Mars in ensuring spindle integrity, the contribution of the N-terminal region to MT stabilization is more important than the effect of the C-terminal region on dephosphorylation of dTACC.

To identify proteins that co-purify with GFP-Mars from embryos, we performed mass spectrometry. We identified PP1 and Msps to be associated with GFP-Mars, as reported previously [10]. However, we did not find dTACC protein associated with GFP-Mars, further questioning the relevance of dTACC dephosphorylation by Mars and PP1 for spindle integrity. The most abundant protein identified in the immune complex except Mars itself was importin beta (data not shown). How the function of Mars is regulated by importin beta is an interesting question that needs further investigation.

In our previous study we proposed a functional homology between *Drosophila* Mars and mammalian NuMA [11]. A recent study of NuMA in mouse embryogenesis found that after inactivation of NuMA, spindles initially formed with MTs focussed at centrosomes. However, subsequent to spindle assembly and upon generation of spindle forces, centrosome-spindle attachment was uncoupled. Kinetochore fibers defocussed and centrosomes failed to maintain and reestablish connection to the spindle. Surprisingly, chromosome segregation was largely intact [21]. These findings are very similar to what we have reported before for *mars* mutant phenotypes [11], which further confirms our hypothesis that Mars and NuMA may be functional homologs. However, Mars does not have a long coiled-coil domain like NuMA, which is required for its dimerization [22], [23]. Other MT stabilizers have also been reported to crosslink MTs via dimeric or oligomeric complexes such as motor proteins KLP61F [24], Ncd [3] and the non-motor protein Ase1 [25]. It will be very interesting to investigate whether Mars also executes the crosslinking in a dimeric or oligomeric complex.

## Supporting Information

Figure S1 **GFP-N-Mars expression stabilizes the mitotic spindle in embryos.** Wild type embryos were mixed with embryos overexpressing GFP-N-Mars. Embryos were treated with 0.15 µM demecolcine before fixation and stained with tubulin antibody (red), GFP antibody (green) and DAPI (turquoise). Scale bar is 5 µm.
(TIF)

Movie S1 **Subcellular localization of GFP-N-Mars.** A *Drosophila* embryo expressing GFP-N-Mars at the syncytial blastoderm stage was imaged live by confocal microscopy.
(MOV)

Movie S2 **Spindle fusion upon overexpression of GFP-N-Mars.** The movie shows the fusion of two adjacent mitotic spindles in a *Drosophila* embryo overexpressing GFP-N-Mars at the syncytial blastoderm stage.
(MOV)

Movie S3 **Subcellular localization of GFP-C-Mars.** A *Drosophila* embryo expressing GFP-C-Mars at the syncytial blastoderm stage was imaged live by confocal microscopy.
(MOV)

Movie S4 **Nuclear fusion upon overexpression of GFP-C-Mars.** The movie shows the fusion of two adjacent nuclei in a *Drosophila* embryo overexpressing GFP-C-Mars at the syncytial blastoderm stage.
(MOV)
