# Supplementary figures and images for: The Drosophila Microtubule-Associated Protein Mars Stabilizes Mitotic Spindles by Crosslinking Microtubules through Its N-Terminal Region
Source: PLoS One. 2013 Apr 4;8(4):e60596. doi: 10.1371/journal.pone.0060596 (PMC3617137; doi:10.1371/journal.pone.0060596)

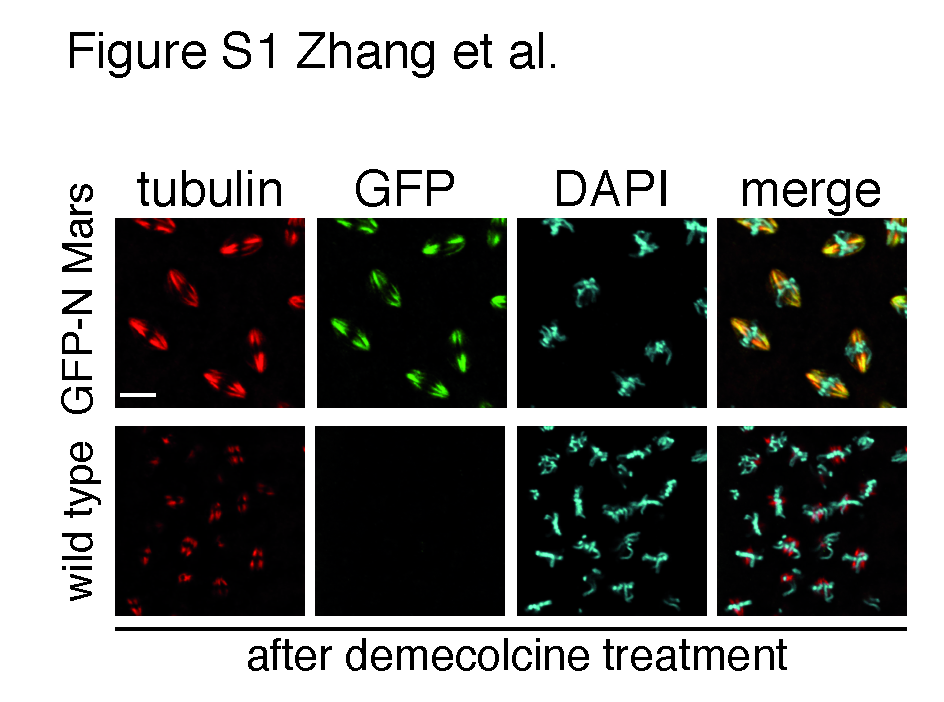

Supplement: Figure S1 — GFP-N-Mars expression stabilizes the mitotic spindle in embryos. Wild type embryos were mixed with embryos overexpressing GFP-N-Mars. Embryos were treated with 0.15 µM demecolcine before fixation and stained with tubulin antibody (red), GFP antibody (green) and DAPI (turquoise). Scale bar is 5 µm. (TIF) [file pone.0060596.s001.tif]
